# Protective Effects of Long-Term Usage of Cyclo-Oxygenase-2 Inhibitors on Colorectal Cancer in Genetically Predisposed Individuals and Their Overall Effect on Prognosis: A Systematic Review

**DOI:** 10.7759/cureus.41939

**Published:** 2023-07-15

**Authors:** Sri Harsha Narayana, Ujala Mushtaq, Basim Shaman Ameen, Chuhao Nie, Daniel Nechi, Iqra J Mazhar, Mohamed Yasir, Saba Sarfraz, Gandhala Shlaghya, Safeera Khan

**Affiliations:** 1 Research, California Institute of Behavioral Neurosciences & Psychology, Fairfield, USA; 2 Internal Medicine, California Institute of Behavioral Neurosciences & Psychology, Fairfield, USA

**Keywords:** non-steroidal anti-inflammatory drugs (nsaid), protective effects, prognosis, selective cox-2 inhibitors, cox-2 inhibitors, therapeutic use, prevention, colorectal cancer

## Abstract

Colorectal cancer (CRC) is a major global health concern, accounting for significant cancer-related morbidity and mortality worldwide. Despite advancements in early detection and treatment modalities, the prevention of CRC remains a critical goal. Cyclo-oxygenase-2 (COX-2) is an inducible enzyme involved in the production of pro-inflammatory prostaglandins, which play a crucial role in various cellular processes, including inflammation, cell proliferation, apoptosis, and angiogenesis. Elevated COX-2 expression has been consistently observed in colorectal tumors, indicating their role in the pathogenesis of cancer. COX-2 inhibitors, such as celecoxib and rofecoxib, have been studied as potentially effective treatment modalities due to their ability to decrease prostaglandin levels, which are generally higher in cancer patients. Aberrant prostaglandin production is linked to the adenoma-carcinoma sequence, during which adenomas turn dysplastic and accumulate enough damage to become malignant. COX-2 inhibitors have also been shown to modulate various signaling pathways involved in CRC development, such as wingless-related integration site/β-catenin (Wnt/β-catenin), mitogen-activated protein kinase (MAPK), and phosphoinositide-3-kinase-protein kinase B/Akt (PI3K/Akt) pathways. This systematic review aimed to evaluate the protective effects of long-term usage of COX-2 inhibitors on CRC in genetically predisposed individuals and their overall effect on the prognosis of the disease. The researchers conducted a systematic review following the Preferred Reporting Items for Systematic Reviews and Meta-Analyses (PRISMA) 2020 guidelines and collected data from several databases, including PubMed, PubMed Central, Cochrane Library, and Web of Science. The search strategy combined keywords related to CRC, COX-2 inhibitors, protective effects, and prognosis. They identified 1189 articles and shortlisted 26 full-text articles that met the eligibility criteria. Quality assessment tools, such as the Assessment of Multiple Systematic Review (AMSTAR) for systematic reviews, the Cochrane bias assessment tool for randomized control trials, the scale for the assessment of narrative review articles (SANRA) checklist for narrative reviews, and the Joanna Briggs Institute (JBI) tool for cross-sectional studies and case reports, are used. This review's conclusions will assist in determining the effectiveness of COX-2 inhibitors to prevent CRC. This review may also contribute to developing guidelines for clinicians to manage genetically predisposed individuals with CRC. Furthermore, the results of this review will shed light on the potential of COX-2 inhibitors as a preventive measure against CRC in genetically predisposed individuals.

## Introduction and background

The third most prevalent cancer overall and the most common gastrointestinal (GI) cancer in the United States of America (USA) is colorectal cancer (CRC). Additionally, it is listed as the third-leading cause of cancer-related morbidity and mortality in the USA [1]. A significant proportion of CRC cases are associated with genetic predisposition. About five percent of new-onset CRC cases have a genetic component associated with carcinogenesis [2,3]. It affects men more than women and is more common in developed countries, such as the USA, due to lifestyle changes, including a lack of exercise and dietary habits predominantly involving meat and alcohol [4]. Fruits and dietary fiber are important components of a healthy diet and have been associated with various health benefits, including potential protective effects against CRC [5,6]. Eating fruit and consuming enough fiber may reduce the risk of CRCs, while smoking may raise the risk of colorectal tumors in populations at risk [5]. The high content of antioxidants, vitamins, minerals, and dietary fiber in fruits may contribute to their protective effects by reducing oxidative stress, promoting bowel regularity, and providing essential nutrients that support overall GI health. Studies have consistently demonstrated a positive association between obesity and CRC [2].

Most tissues have a constitutive expression of cyclo-oxygenase-1 (COX-1), which produces prostaglandins in physiological quantities. On the other hand, cyclo-oxygenase-2 (COX-2) is routinely expressed in activated macrophages and is raised in malignant cells to support angiogenesis in tumors. It is also highly upregulated after exposure to growth factors or inflammatory stimuli [7]. In CRC, COX-2 overexpression has been linked to more aggressive characteristics, such as increased depth of invasion, a higher tumor stage, and lymph node metastasis. While several treatment options exist for CRC, there is still room for improvement [8]. Adenomas typically precede CRC in most instances. Most colorectal malignancies are believed to arise from adenomas through the adenoma-carcinoma sequence [1,9]. Initially, a small adenoma forms, becoming dysplastic and eventually accumulating enough genomic damage to become an aggressive cancer. This process may take many years to complete and is likely associated with aberrant prostaglandin production [9]. Additionally, overexpression of COX-2 mRNA (messenger ribonucleic acid) contributes to the prevalence of CRC [10].

Researchers currently focus on the prevention and regression of adenomas, often utilizing NSAIDs such as aspirin to reduce the occurrence of CRC precursors. However, NSAIDs are associated with gastritis and GI bleeding [11-14]. COX-2 inhibitors have been shown to have chemopreventive properties in both preclinical and clinical studies [15-18]. Consequently, studies have been conducted to evaluate how specific COX-2 inhibitors, such as celecoxib and rofecoxib, affect the growth of adenomas and assist in disease management [19-24]. Elevated prostaglandin levels, particularly prostaglandin E2 (PGE2), are commonly observed in individuals with cancer. Prostaglandin synthesis involves the catalytic action of cyclo-oxygenase-1 (COX-1) and COX-2 enzymes, and the upregulation of COX-2 results in the release of prostaglandins, which significantly contribute to tumor cell proliferation. Lynch syndrome and familial adenomatous polyposis (FAP) are two genetic conditions associated with an increased risk of developing CRC [2,5]. The adenomatous polyposis coli (APC)-catenin pathway is activated, B-cell leukemia/lymphoma 2 protein (bcl-2 protein) activity is increased, increased levels of vascular endothelial growth factor (VEGF) are produced, and Fas-induced apoptosis is decreased through the action of COX-2 [15].

In this systematic review, we aimed to evaluate the protective effects of long-term usage of COX-2 inhibitors in genetically predisposed individuals for CRC and their overall impact on the prognosis of the condition.

## Review

Methods

Search Sources and Search Strategy

We used the Preferred Reporting Items for Systematic Reviews and Meta-Analyses (PRISMA) 2020 guidelines to conduct the research. Our search includes databases such as PubMed, PubMed Central, Medline, Cochrane Library, and Web of Science to collect the relevant information. We combined keywords such as CRC, COX2 inhibitors, prognosis, and protective effects to search databases. However, PubMed certain search strategy was developed for PubMed's MeSH database: "Colorectal Neoplasms/drug therapy"[Mesh] OR "Colorectal Neoplasms/prevention and control"[Mesh] OR "Colorectal Neoplasms/therapy"[Mesh]) AND "Cyclooxygenase 2 Inhibitors/therapeutic use"[Mesh]).

Table 1 displays the articles found in each database after the search method.

**Table 1:** Search Strategy.

KEYWORDS/SEARCH STRATEGY	DATABASE	RESULTS
Colorectal cancer and cox2 inhibitors, colorectal cancer or cox 2 inhibitors	PubMed	651
:( "Colorectal Neoplasms/drug therapy"[Mesh] OR "Colorectal Neoplasms/prevention and control"[Mesh] OR "Colorectal Neoplasms/therapy"[Mesh] ) AND "Cyclooxygenase 2 Inhibitors/therapeutic use"[Mesh])	PubMed MeSH	157
Colorectal cancer and cox2 inhibitors, colorectal cancer or cox 2 inhibitors	PubMed Central	313
Colorectal cancer and cox2 inhibitors, colorectal cancer or cox 2 inhibitors	Cochrane Library	38
Colorectal cancer and cox2 inhibitors, colorectal cancer or cox 2 inhibitors	Web of Science	30

Inclusion and Exclusion Criteria

We selected the articles published in the last 25 years, which included articles written in English or full-text articles available in English. We included only human participants of all age groups. We did not include pregnant women; those articles whose full text was not retrieved were excluded; grey literature was also excluded.

Process of Selection

We collected the articles using the keyword search strategy, transferred them to the endnote, and recovered the duplicates. We screened each article with titles, abstracts, and eligibility criteria such as inclusion and exclusion criteria and assessed for full-text articles, and then the articles were selected accordingly.

Quality Check of Articles

All the shortlisted articles are screened for quality checks using the relevant quality assessment tools. Systematic reviews were checked by the assessment of multiple systematic review (AMSTAR) tools, randomized controlled trials were checked by the Cochrane bias assessment tool, narrative reviews by scale for the quality assessment of narrative review articles (SANRA) checklist, and cross-sectional studies and case reports are assessed by the Joanna Briggs Institute (JBI) tool.

Results

**Figure 1:**
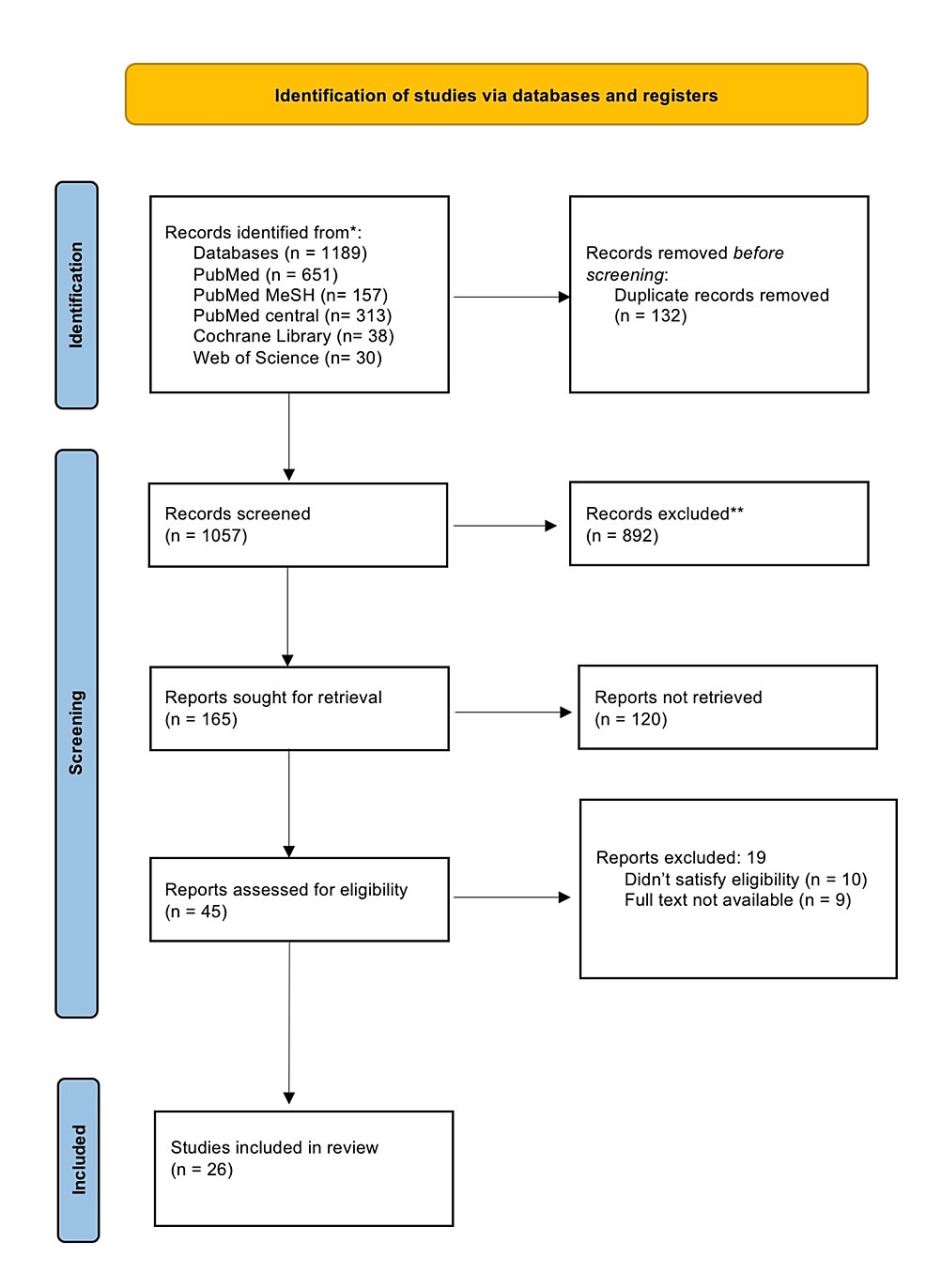
PRISMA Flowchart of the Selected Articles. PRISMA: Preferred Reporting Items for Systematic Reviews and Meta-Analyses.

We identified a total of 1189 articles using the databases. From them, we removed 132 duplicates through endnote, and later the articles were screened by looking into the titles, abstracts, and full-text articles, and of them, 45 articles were shortlisted. These shortlisted full-text articles were assessed for eligibility criteria and were undergone quality checks using the quality assessment tools, and finally 26 articles were finalized for the review. Figure 1 shows the selection process of studies in detail.

Outcome Measures

This study systematically reviewed data from multiple studies to evaluate the effects of long-term usage of COX-2 inhibitors on CRC in genetically predisposed individuals. It compared the incidence rates of CRC and the presence and size of adenomas in individuals treated with COX-2 inhibitors versus control or placebo groups. Additionally, the study analyzed the characteristics of colorectal tumors, including stage, invasion depth, metastasis, and differentiation, concerning COX-2 inhibitors. It also investigated the expression levels of COX-2 in CRC tissue and adjacent normal mucosa. This study aimed to provide insights into the potential preventive role of COX-2 inhibitors.

Study Characteristics

We conducted a comprehensive review that included 26 research papers. Four randomized controlled trials, three cross-sectional studies, two case-control studies, 10 narrative reviews, three systematic reviews and meta-analyses, one systemic review and one meta-analysis, one case report, and one clinical trial collectively comprise the research studies that were selected.

The sample sizes of the included studies were reported, detailing the number of participants in each study group, such as the COX-2 inhibitor group, control group, or placebo group. The duration of the studies, including the length of follow-up or treatment with COX-2 inhibitors, was also provided. Participant characteristics, such as age, gender distribution, genetic predisposition to CRC, and inclusion/exclusion criteria, were described to assess the generalizability of the findings. The specific COX-2 inhibitors used, including dosage, frequency, and duration of treatment, were outlined. The control groups employed, such as placebo, no-treatment, or alternative treatment groups, were mentioned. The statistical methods used for data analysis, encompassing descriptive and inferential statistics and measures of effect size, were reported to facilitate result interpretation and determine the significance of the findings.

The articles were assessed for eligibility using different quality appraisal tools. Table 2 below shows the quality appraisal for the case reports, Table 3 for the narrative reviews, Table 4 for cross-sectional studies, Table 5 for systematic reviews and meta-analyses, and Table 6 for studies relevant to this review.

**Table 2:** Quality Appraisal Using the JBI Tool for Case Reports. JBI: Joanna Briggs Institute

STUDY	Q1	Q2	Q3	Q4	Q5	Q6	Q7	Q8	Q9
Dominic et al. 2020 [1]	YES	YES	YES	YES	YES	YES	YES	YES	GOOD

**Table 3:** SANRA Checklist for Narrative Reviews. SANRA: Scale for the quality assessment of narrative review articles.

STUDY	Q1	Q2	Q3	Q4	Q5	Q6	SUM SCORE
Tomita et al. 2021 [3]	2	2	1	2	2	2	11
Ganduri et al. 2022 [4]	2	2	0	2	2	2	10
Potter 1999 [6]	2	2	0	2	2	2	10
Luo et al. 2005 [7]	2	2	1	2	2	2	11
Sheng et al. 2020 [8]	2	2	0	2	2	2	10
Ranger 2014 [9]	2	2	0	2	2	2	10
Chan et al. 2012 [12]	2	2	1	2	2	2	11
Castellone et al. 2006 [15]	2	2	0	2	2	2	10
Wen et al. 2020 [22]	2	2	1	2	2	2	11
Gonzalez-Angulo et al. 2002 [24]	2	2	0	2	2	2	10

**Table 4:** JBI Tool for Cross-Sectional Studies. JBI: Joanna Briggs Institute

STUDY	Q1	Q2	Q3	Q4	Q5	Q6	Q7	Q8
Roelofs et al. 2014 [10]	YES	YES	NOT APPLICABLE	YES	UNCLEAR	UNCLEAR	YES	YES
Venkatachala et al. 2017 [14]	YES	YES	NOT APPLICABLE	YES	UNCLEAR	UNCLEAR	YES	YES
O'Malley et al. 2022 [25]	YES	YES	N0T APPLICABLE	YES	UNCLEAR	UNCLEAR	YES	YES

**Table 5:** AMSTAR Checklist for Systematic Reviews and Meta-analysis. AMSTAR: A measurement tool to assess systematic reviews.

AMSTAR criteria	Guirguis-Blake et al. 2020 [11]	Harewood et al. 2021 [13]	Ma et al. 2023 [20]	Ye et al. 2022 [26]
Did the research questions and inclusion criteria for the review include the components of PICO?	NO	NO	NO	NO
Was a “priori” design implemented?	YES	YES	YES	YES
Did the review authors explain their selection of the study designs for inclusion in the review?	YES	YES	YES	YES
Did the review authors use a comprehensive literature search strategy?	YES	YES	YES	YES
Did the review authors perform study selection in duplicate?	YES	YES	YES	YES
Did the review authors perform data extraction in duplicate?	UNCERTAIN	YES	YES	YES
Did the review authors provide a list of excluded studies and justify the exclusions?	NO	YES	YES	YES
Did the review authors describe the studies included in adequate detail?	YES	YES	YES	YES
Did the review authors use a satisfactory technique for assessing the risk of bias in individual studies that were included in the review?	YES	YES	YES	YES
Did the review authors report on the sources of funding for the studies included in the review?	YES	YES	YES	YES
If a meta-analysis was performed, did the authors use appropriate methods to statistically combine results?	NOT APPLICABLE	YES	YES	YES
If a meta-analysis was performed, did the review authors assess the potential impact of risk of bias in individual studies on the results of the meta-analysis or other evidence synthesis?	NOT APPLICABLE	YES	YES	YES
Did the review authors account for the risk of bias in individual studies when interpreting/discussing the results of the review?	NO	YES	YES	UNCERTAIN
Did the review authors provide a satisfactory explanation for and discussion of any heterogeneity observed in the results of the review?	YES	UNCERTAIN	YES	YES
If they performed quantitative synthesis, did the review authors carry out an adequate investigation of publication bias (small study bias) and discuss its impact on the results of the review?	NO	NO	YES	YES
Did the review authors report any potential sources of conflict of interest, including any funding they received for conducting the review?	YES	YES	YES	YES
Total score (out of 16)	11/16	12/16	15/16	14/16
Overall quality	68.75%	75%	93.75%	87.5%

**Table 6:** Relevant Studies Exploring the Protective Effects of the Long-Term Usage of Cyclo-Oxygenase-2 Inhibitors on Colorectal Cancer and Their Overall Effect on Prognosis of the Condition. COX: cyclo-oxygenase, COX-1: cyclo-oxygenase-1, COX-2: cyclo-oxygenase-2, NSAID: non-steroidal anti-inflammatory drugs, PGE2: prostaglandin e2, PGI2: prostaglandin i2, GADPH: glyceraldehyde-3-phosphate dehydrogenase, PIK3CA: phosphatidylinositol, mRNA: messenger ribonucleic acid, CI: confidence interval, OR: odds ratio, HR: hazard ratio, RCT: randomized controlled trial, ORR: objective response rate, OS: overall survival, FOLFIRI: folinic acid/fluorouracil/irinotecan regimen, VEGF: vascular endothelial growth factor, CXCL5: C-X-C motif chemokine ligand 5, FASL: FS-7-associated surface antigen ligand, sFASL: soluble FS-7-associated surface antigen ligand.

Authors and year of publication	Type of study	Purpose of study	Number of participants	Results	Conclusions
Dominic et al. 2020 [1]	Case Report	Following a synchronous primary colorectal cancer, the presentation of chemotherapeutically resistant metachronous colorectal cancer due to aberrant chromosomal instability	1	-	For patients with aberrant partial chromosomal instability with chemotherapeutically resistant metachronous colorectal cancer, there is a need for more specialized methods of diagnosis, treatment, and follow-up.
Lazzeroni et al. 2021 [2]	Meta-analysis	Association of obesity with colorectal cancer in Lynch syndrome.	5131	Obese men had a twofold increased risk of colorectal cancer compared to men who are not obese (SRR = 2.09; 95% CI = 1.23-3.55, I^2^ = 33%). For women, there was no discernible rise in risk from obesity. For those with an MLH1 mutation, the probability of developing colorectal cancer due to obesity increased by 49% (SRR = 1.49; 95% CI = 1.11-1.99, I^2^ = 0%).	There is a positive association between obesity and colorectal cancer in men. MLH1 mutation is also positively associated with the development of colorectal cancer.
Tomita et al. 2021 [3]	Narrative Review	Guidelines for clinical management of hereditary colorectal cancer.	0	-	Guidelines for clinical management of colorectal cancer developed.
Ganduri et al. 2022 [4]	Narrative Review	Role of COX inhibitors in colorectal cancer.	0	When compared to sporadic forms, COX inhibition has shown statistically significant results in lowering the risk of familial colorectal cancer.	COX inhibitors can be used for chemoprevention of colorectal cancer.
Diergaarde et al. 2007 [5]	Case-Control Study	Role of environmental factors in colorectal carcinogenesis.	248	Fruit intake was substantially inversely related to having ever had a colorectal tumor diagnosis (OR (95% CI) for highest versus lowest tertile = 0.4 (0.2-0.9); P trend =0.03. Dietary fiber intake showed an inverse correlation that was only marginally statistically significant (0.5(0.2-1.0); P trend = 0.06).	In people with HNPCC, eating fruit and getting enough dietary fiber may reduce the risk of colorectal cancers, whereas smoking may raise the risk of HNPCC-related colorectal tumors.
Potter et al. 1997 [6]	Narrative Review	Molecular biology and epidemiology of colorectal cancer.	0	By genetically selecting a group of cells that do not show microsatellite instability, NSAIDs (including aspirin) may directly decrease the HNPCC-associated mutator phenotype. Even individuals who are genetically at high risk could benefit greatly from using these NSAIDs.	NSAIDs may reduce the development of colorectal cancer.
Luo et al. 2005 [7]	Narrative Review	Role of COX-2 in inhibitors in colorectal cancer management.	0	COX-2 inhibitors act by decreasing the production of prostaglandin E2, which plays a key role in colorectal carcinogenesis.	COX-2 inhibitors can be used for chemoprevention in colorectal cancer.
Sheng et al. 2020 [8]	Narrative Review	Role of COX-2 in colorectal cancer.	0	Specific COX-2 inhibitors, such as NSAIDs, may reduce the risk and improve the prognosis of carcinogenesis of several forms of cancer, including colon cancer, by inhibiting PGE2 synthesis. In order to successfully use COX-2 inhibitors in clinical applications to colorectal malignancies and other types of cancer as well, we must work to develop a protocol.	The majority of the cells in the mesenchyme are fibroblasts. COX-2 expression, which is well established as a key component in colorectal carcinogenesis, is abundantly expressed by fibroblasts from non-neoplastic colorectal tissue. It is vital to understand that, the regulation of the COX-2 gene is essential for the management of this condition.
Ranger et al. 2014 [9]	Narrative Review	Current concepts in colorectal cancer prevention with COX inhibitors.	0	In contrast to the previous belief that it takes greater doses of aspirin which means more than 1 g daily, sustained low-dose NSAID/aspirin administration was related to a 30%–50% reduction in polyp development, cancer incidence, and mortality.	It's possible that COX-1 activity in activated platelets serves as a signal to induce COX-2 expression. This may help to explain why aspirin and NSAIDs have chemopreventive effects even at doses that make it impossible to reduce COX-2 expression in nucleated cells. The longer-term follow-up may have finally revealed this impact. Permanent platelet COX-1 inactivation may prevent COX-2 up-regulation in neighboring cell types in the intestinal mucosa at mucosal damage sites. Aspirin may potentially have effects through COX-2-independent mechanisms, such as those linked to phosphatidylinositol 3-kinase (PI3KCA). Aspirin consumption in this group following colon cancer resection was associated with a significant improvement in survival, suggesting that tumors with mutant PI3KCA may be more sensitive to chemoprevention with aspirin.
Roelofs et al. 2014 [10]	Cross-Sectional Study	Over-expression of COX-2 mRNA levels in colorectal cancer.	60	In contrast to the neighboring matched normal colorectal mucosa samples, COX-2 mRNA levels were overexpressed in 80%, 70%, and 40% of the colorectal tumor tissues, respectively, normalized with regard to tissue weight or the mRNA levels of the housekeeping genes B2M or GAPDH. The highest tumor/normal mRNA COX-2 ratios (mean ratio = 21.6) were seen when expressed per mg of tissue. Mean tumor/normal ratios were 16.1 and 7.5 when compared to the housekeeping genes B2M or GAPDH, respectively.	In contrast to paired adjacent normal colorectal mucosa, levels of COX-2 mRNA are found to be overexpressed in over 80% of colorectal tumors, suggesting a function for COX-2 as a potential biomarker for cancer risk. COX-2 inhibitors may be useful in the chemoprevention of colon cancer.
Guirguis-Blake et al. 2020 [11]	Systematic Review and Meta-analysis	Aspirin's role in preventing cardiovascular events and colorectal cancer.	134870	Major cardiovascular disease events were significantly reduced by low-dose aspirin (odds ratio (OR) = 0.90, 95% CI = 0.85-0.95); 11 RCTs (n = 134 470); I2 = 0%; range in absolute effects = 2.5% to 0.1%). Individual cardiovascular disease outcomes showed statistically significant results with comparable magnitudes of benefit. There was little information from trials on the advantages of colorectal cancer, and the results were very diverse depending on how long the follow-up lasted.	small absolute risk reductions in major cardiovascular disease events and minor absolute increases in major bleeding were linked to low-dose aspirin use. Results for colorectal cancer were less consistent and highly inconsistent.
Chan et al. 2011 [12]	Narrative Review	Role of aspirin in chemoprevention of colorectal cancer.	0	Aspirin use for an extended period of time may be considered to prevent colorectal cancer. However, there is a lack of agreement over how long-term aspirin use balances risks and benefits, especially in low-risk populations.	Long-term aspirin use can be considered for colorectal cancer prevention.
Harewood et al. 2021 [13]	Systematic Review + Meta-analysis	Risk of colorectal cancer with medication use.	1910823	A statistically significant reduction in the incidence of proximal colon cancer was seen for aspirin use, but there was also strong evidence of between-study heterogeneity (I^2^ = 67.1%, p = 0.002). The pooled estimates for both aspirin and non-aspirin NSAID use were not statistically significant, but there was some indication of study heterogeneity for any non-steroidal anti-inflammatory drugs use (I^2^ = 64.4%, p = 0.060).	The use of NSAIDs and proximal colon cancer is inversely correlated.
Venkatachala et al. 2017 [14]	Cross-Sectional Study	Clinicopathological features and COX-2 expression in colorectal carcinoma.	65	High COX-2 expression was seen in about 56.6% of well-differentiated carcinomas, 66.6% of moderately differentiated carcinomas, and 100% of poorly differentiated carcinomas. The depth of invasion (p = 0.021), tumor stage (p = 0.05), frequency of lymph node metastasis, and degree of differentiation were all correlated with COX-2 overexpression.	The use of COX-2 inhibitors as an adjunct to chemotherapy and radiation therapy may be justified by the correlation between COX-2 overexpression and increasing stage and depth of invasion.
Castellone et al. 2006 [15]	Narrative Review	COX-2 and colorectal cancer chemoprevention: the b-catenin connection.	0	NSAID use is linked to a lower incidence of colorectal cancer, however long-term nonselective NSAID use is linked to gastrointestinal damage because it inhibits the COX-1 isoform, a crucial housekeeping gene for the digestive tract. The majority of work has gone on creating particular COX-2 enzyme inhibitors. Unfortunately, recent long-term studies have demonstrated that several COX-2 selective medications can raise cardiovascular morbidity. These side effects of long-term COX-2 inhibition are mostly caused by the overall suppression of prostanoid production, which also results in the decreased release of anti-thrombotic prostaglandins such PGI2 and tumor-promoting PGE2. Inhibiting prostaglandin E synthase is one potential method for selectively blocking PGE2. Compared to COX inhibitors, selective inhibitors of this enzyme, which is overexpressed in malignancies, may be more efficient and produce greater therapeutic ratios. Additionally, modifying the proteins linked to the Gs-catenin and NR4A2 pathways with particular molecular inhibitors may offer novel chemopreventive methods for colorectal cancer.	Numerous COX-2 selective drugs have been shown in long-term research to increase cardiovascular morbidity. These negative effects of long-term COX-2 inhibition are mostly brought about by the general suppression of prostanoid production, which also causes a reduction in the release of tumor-promoting PGE2 and anti-thrombotic prostaglandins such PGI2. Selective inhibitors of PGE2, which are overexpressed in malignancies, could be more effective than COX inhibitors and result in a higher therapeutic advantage.
Steinbach et al. 2000 [16]	Randomized Controlled Trail	The effect of celecoxib in familial adenomatous polyposis.	77	After six months, the individuals taking 400 mg of celecoxib twice a day had 28.0% fewer colorectal polyps on average and 30.7% less polyps overall, as opposed to 4.5% and 4.9%, respectively, in the placebo group. A group of endoscopists who saw the videotapes confirmed that the group taking 400 mg twice a day had less colorectal polyposis overall. The reductions were 11.9% and 14.6%, respectively, in the group taking 100 mg of celecoxib twice daily. The frequency of adverse events was similar amongst the groups.	The number of colorectal polyps is significantly decreased in patients with familial adenomatous polyposis after six months of twice-daily treatment with 400 mg of celecoxib, a COX-2 inhibitor.
Arber et al. 2006 [17]	Randomized Controlled Trail	The use of celecoxib to prevent colorectal adenomatous polyps.	1561	264 and 270, respectively, of the 840 participants in the celecoxib group and the 557 subjects in the placebo group who were included in the efficacy analysis, were found to have at least one adenoma at either the first year, the third year, or both. Adenomas were found at a cumulative rate of 33.6% in the celecoxib group and 49.3% in the placebo group (relative risk = 0.64; 95% CI = 0.56-0.75; p = 0.001). When advanced adenomas were cumulatively found through year three, the celecoxib group had a rate of 5.3% and the placebo group saw a rate of 10.4% (relative risk = 0.49; 95% CI = 0.33-0.73; p = 0.001).	Within three years of polypectomy, the usage of 400 mg of celecoxib once a day significantly reduced the occurrence of colorectal adenomas.
Bresalier et al. 2006 [18]	Clinical Trail	Rofecoxib-related cardiovascular incidents in a colorectal adenoma chemoprevention trial.	2586	46 individuals in the rofecoxib group encountered a confirmed thrombotic event over the course of 3,059 patient-years of follow-up, as opposed to 26 individuals in the placebo group during 3,327 patient-years of follow-up. After 18 months of treatment, the increased relative risk became obvious; for the first 18 months, the incident rates were similar in the two groups. The primary conclusion is that the rofecoxib group experienced more myocardial infarctions and ischemic cerebrovascular events.	In patients with a history of colorectal adenomas, the use of rofecoxib has been associated with an elevated cardiovascular risk.
Baron et al. 2006 [19]	Randomized Controlled Trail	Rofecoxib for the chemoprevention of colorectal adenomas: A randomized trial.	2587	Adenoma recurrence was less likely in participants using rofecoxib than in those who were given a placebo. Additionally, rofecoxib reduced the probability of advanced adenomas (p = 0.01). The first year had a stronger chemopreventive effect than the following two years. Rofecoxib has been associated with higher chances of major upper gastrointestinal events and serious thrombotic cardiovascular events, as was previously reported.	Rofecoxib considerably decreased the risk of colorectal adenomas in this randomized control trail; however, it also caused extremely harmful side effects.
Ma et al. 2023 [20]	Systematic Review + Meta Analysis	Chemoprevention of colorectal cancer in both high-risk and general populations.	278,694	There were 32 randomized controlled studies with 278,694 participants that compared 13 various therapies. In comparison to placebo, COX inhibitors significantly decreased the risk of colorectal adenoma, advanced adenoma, and metachronous adenoma in six trials totaling 5,486 people, 4,723 participants in four trials, and 5,258 persons in five trials. In six trials comprising 7,109 people, COX inhibitors also markedly elevated the incidence of severe adverse outcomes. In comparison to placebo, other therapies such as aspirin, folic acid, UDCA, vitamin D, and calcium did not lower the risk of colorectal adenoma in the general or high-risk populations.	The existing data did not support the routine use of COX inhibitors for the prevention of colorectal adenoma when benefits and risks were balanced. There is still a need for more proof of the benefits of low-dose Aspirin for chemoprevention of colorectal adenoma.
Harris et al. 2008 [21]	Case-Control Study	Reductions in the risk of colon cancer by selective and nonselective COX-2 inhibitors.	978	Results revealed that using selective COX-2 inhibitors, regular aspirin, and ibuprofen or naproxen significantly reduced risk. Acetaminophen, a substance with very little COX-2 action, and low-dose aspirin (81 mg) had no discernible effect on colon cancer risk.	Both selective and non-selective COX-2 inhibitors significantly reduce the risk of colon cancer.
Wen et al. 2020 [22]	Narrative Review	Role of celecoxib in colorectal cancer.	0	Through a variety of molecular pathways, celecoxib can inhibit the growth of tumors. It was discovered that celecoxib might increase mitochondrial oxidation, activate the mitochondrial apoptosis process, promote the endoplasmic reticulum stress process, and induce autophagy in tumor cells.	Celecoxib can prevent the growth of tumors and reduce the development of drug resistance.
Mostafa et al. 2022 [23]	Randomized Controlled Trail	Role of celecoxib as adjuvant therapy in colorectal cancer.	54	When compared to the FOLFIRI arm, the celecoxib/FOLFIRI-combined intervention significantly increased ORR (p= 0.001). The VEGF, CXCL5, and sFASL serum levels in the celecoxib/FOLFIRI arm were considerably lower than those in the FOLFIRI arm (p = 0.001), whereas the sFAS serum level and sFAS/FASL ratio were significantly higher (p = 0.001). Additionally, when compared to the FOLFIRI arm, the celecoxib/FOLFIRI arm had significantly greater progression-free survival and one-year overall survival.	Patients with metastatic colorectal cancer may benefit from a safe and effective synergetic treatment that combines celecoxib and chemotherapy.
Gonzalez-Angulo et al. 2002 [24]	Narrative Review	COX-2 inhibitors and colon cancer.	0	For the prevention and treatment of colorectal cancer, selective COX-2 inhibitors may be useful anticancer drugs.	Selective COX-2 inhibitors may be effective anticancer medications for the diagnosis, prognosis, and treatment of colorectal cancer. But there are still many unsolved questions regarding the effectiveness, safety, and mechanisms of action. A thorough understanding of COX-2 inhibitors' mechanism of action is necessary for the creation of highly effective anticancer drugs. The few factors listed below make this difficult. As an example, sulindac sulfone, which does not inhibit COX, prevents colorectal cancer in experimental animals, posing the prospect that COX inhibitors' ability to treat cancer may not be based on their ability to inhibit COX. Epidemiologic investigations and well-planned randomized clinical trials should assist in identifying medications with proven clinical use for cancer prevention and treatment once the efficacy and safety of the experimental drug in issue have been demonstrated in the animal models.
O'Malley et al. 2022 [25]	Cross-Sectional Study	Willingness for genetic testing and use of non-steroidal anti-inflammatory drugs in colorectal cancer survivors.	273	83% of colorectal cancer survivors expressed interest in genetic testing. The study revealed genomic testing interest was associated with being uncoupled (odds ratio (OR) = 4.11; 95% confidence interval (CI) = 1.49-11.35), low income (OR = 0.35, 95% CI = 0.14-0.88), smoking history (OR = 0.35, 95% CI: 0.14-0.90), low health literacy (OR = 0.33, 95% CI = 0.07-1.43) and moderate health literacy (OR = 0.26, 95% CI = 0.11-0.61), and personal colorectal cancer risk worry (OR = 2.86, 95% CI = 1.63-5.02, p = 0.0002). Age (OR = 1.05, 95% CI = 1.01-1.10) and a history of cardiovascular disease (OR = 2.42, 95% CI = 1.23-4.73, p = 0.010) were also related to aspirin use.	The majority of cancer survivors showed interest in genomic testing and the majority of CRC survivors who weren't already aspirin/NSAID users were open to using these medications to reduce their chance of developing cancer.
Ye et al. 2022 [26]	Systematic Review + Meta-analysis	The safety and efficacy of using celecoxib in addition to conventional cancer therapies.	9655	An improved OS was shown when celecoxib was used in conjunction with adjuvant therapy (HR = 0.850, 95%CI = 0.725-0.996). Additionally, celecoxib plus neoadjuvant therapy enhanced the ORR in conventional cancer therapy, particularly neoadjuvant therapy (overall: RR = 1.13, 95% CI = 1.03-1.23; neoadjuvant therapy: RR = 1.25, 95% CI = 1.09-1.44).	Celecoxib plus adjuvant therapy increases ORR, while celecoxib plus neoadjuvant therapy increases OS. As a result, celecoxib-coupled cancer therapy may be an effective treatment plan.

Discussion

Genetic Predisposition for CRC

Genetic factors are crucial in CRC and FAP. In more than 80% of spontaneous CRCs, mutations occur in the APC tumor suppressor gene [24]. Changes in tumor suppressor genes (e.g., APC, TP53: tumor protein 53) and oncogenes (e.g., KRAS: Ki-ras2 Kirsten rat sarcoma viral oncogene homolog) can cause CRC, while FAP is caused by inherited germline mutations in the APC gene, which is located on chromosome six. Multiple colorectal adenomas, ranging in number from a few polyps to several thousand, grow over time, often starting in childhood. At a mean age of 44 years, at least one of the polyps will develop into cancer if left untreated, about 20 years earlier than the average age at which malignancies in those who do not have the condition first emerge [6]. The interaction with these genetic factors becomes significant when considering COX-2 inhibitors, which selectively block the COX-2 enzyme. COX-2 inhibitors may affect inflammation, cell proliferation, and dysregulated signaling pathways in individuals with specific genetic alterations associated with CRC. Synchronous primary CRC may develop from FAP. This can occasionally result in the inability to achieve remission despite therapy and present as metachronous cancer, requiring close monitoring. Unfortunately, there is no research on the best practices for managing synchronous and metachronous CRCs based on aberrant mutational analyses [1]. In the case of FAP, COX-2 inhibitors show the potential to reduce polyp formation and growth. The response to COX-2 inhibitors may vary based on specific genetic mutations and individual factors. Genetic profiles can optimize treatment strategies for better outcomes in genetically predisposed individuals [2,3,5].

COX: The Housekeeping Enzyme and Its Various Functions

COX-1 and COX-2 are involved in prostaglandin production. Several organs, most notably the glomeruli of the kidney and the cortex of the brain, physiologically and functionally express COX-2 [7]. These enzymes convert arachidonic acid to prostaglandin H2, which is further converted to various prostaglandins, thromboxanes, and prostacyclins [24]. COX-2 is an enzyme that plays an important role in the body's inflammatory response. COX-2 increases the production of prostaglandins, especially PGE2, which plays a major role in inflammation and causes fever during injuries. COX-2 is important in decreasing Fas-induced apoptosis, increasing B-cell leukemia/lymphoma 2 protein (bcl-2) activity, activating the APC-β catenin pathway, and producing a high amount of VEGF. PGE2, produced due to the increased activity of COX-2 in colon cancer, promotes the growth of cancer cells by activating a signaling pathway involving G proteins [15]. This pathway links the prostaglandin EP2 receptor to beta-catenin regulation. 

COX-2 Enzyme in Colorectal Carcinogenesis

Two hereditary diseases linked to a higher chance of developing CRC include Lynch syndrome and FAP. Studies have shown that using COX-2 inhibitors, such as celecoxib, can reduce the incidence of colorectal polyps in individuals with Lynch syndrome and FAP [16,17].

A study by O'Malley et al. is an important initial step toward understanding the potential implementation of genomic testing and targeted non-steroidal anti-inflammatory drug (NSAID) use for CRC recurrence reduction. The majority of CRC survivors cited interest in genomic testing to guide cancer-risk management strategies. Specifically, 83% of CRC survivors expressed interest in genetic testing. The study revealed genomic testing interest was associated with being uncoupled (odds ratio (OR) = 4.11, 95% confidence interval (CI) = 1.49-11.35), low income (OR = 0.35, 95% CI = 0.14-0.88), smoking history (OR = 0.35, 95% CI = 0.14-0.90), low health literacy (OR = 0.33, 95% CI = 0.07-1.43), moderate health literacy (OR = 0.26, 95% CI = 0.11-0.61), and personal CRC risk worry (OR = 2.86, 95% CI = 1.63-5.02, p = 0.0002). Age (OR = 1.05, 95% CI = 1.01-1.10) and a history of cardiovascular disease (OR = 2.42, 95% CI = 1.23-4.73, p = 0.010) were also related to aspirin use [25]. 

The primary target of cytokines such as interleukin-1 (IL-1) and tumor necrosis factor-alpha (TNFα) are fibroblasts from the mesenchymal (stromal) layer. Fibroblasts make up the majority of mesenchymal cells, and fibroblasts from non-neoplastic colorectal tissue are an important source of COX-2 expression, which is well-established as a crucial process in the development of CRC [8]. Aspirin and other NSAIDs have been investigated in numerous recent trials for chemoprevention of CRC [15-24]. It is possible that COX-1 activity in activated platelets serves as a signal to induce COX-2 expression by blocking the release of paracrine lipid and protein mediators that induce COX-2 expression. This may help explain why aspirin and NSAIDs have chemopreventive effects even at doses that make it impossible to reduce COX-2 expression in nucleated cells [9]. However, recent investigations have found that, by precisely targeting the COX-2 enzyme, inhibiting it, and limiting the generation of prostaglandins, COX-2 inhibitors significantly impact inflammation and cancer. This also explains their anti-inflammatory and antipyretic effects. NSAIDs, which also block the COX-1 enzyme, can result in GI side effects such as bleeding and ulcerations due to the loss of mucosal protection effect of prostaglandins. COX-2-specific inhibitors, on the other hand, spare COX-1 and are less likely to cause GI side effects. A study by Harewood et al. showed a protective effect of aspirin usage (OR = 0.80, 95% CI = 0.73-0.89), but no correlations between hormone replacement therapy (RR = 0.92, 95% CI = 0.83-1.02), oral contraceptives (RR = 1.06, 95% CI = 0.98-1.14), or statin use (RR = 0.94, 95% CI = 0.67-1.31) and the incidence of proximal colon cancer compared to never/non-use. There was no correlation between the anti-hypertensives and metformin in either study. However, COX-2 inhibitors have been associated with increased cardiovascular events, such as stroke and heart attack, because the prostaglandins produced by COX-2 have protective effects on the cardiovascular system. A cross-sectional study by Roelofs et al. measured COX-2 mRNA levels in 60 paired samples of non-cancerous tumor tissue and comparable normal colon mucosa taken at least 5 cm away from the tumor [8]. COX-2 mRNA levels are reported to be overexpressed in over 80% of colorectal malignancies when compared with paired adjacent normal colorectal mucosa, implying a function for COX-2 as a possible marker for the likelihood of cancer, whereas COX-2 inhibitors could be useful in colon cancer prevention [10]. 

Several studies have investigated the potential use of COX-2 inhibitors as chemopreventive agents for Lynch syndrome and FAP [16,17]. Steinbach et al. conducted a randomized trial that demonstrated a significant reduction in colorectal polyp burden in FAP patients treated with celecoxib, a COX-2 inhibitor, compared to placebo [16]. It was discovered that celecoxib might increase mitochondrial oxidation, activate the mitochondrial apoptosis process, promote endoplasmic reticulum stress, and promote autophagy to facilitate the apoptosis of tumor cells [22]. A key mechanism for tumor metastasis is angiogenesis. VEGF is a powerful angiogenic factor that affects the growth and survival of endothelial cells, the growth of new blood vessels, and the suppression of the immune response of the host, among other things that help tumors grow. Compared to the folinic acid, fluorouracil, and irinotecan (FOLFIRI) regimen alone, celecoxib and FOLFIRI together caused a statistically significant drop in serum VEGF levels. This finding suggests that celecoxib may have anti-angiogenic properties. The ability of celecoxib to inhibit the COX-2 enzyme with subsequent decreased PGE2, which plays a crucial role in the generation of VEGF, may be the cause of the suppression of angiogenesis translated by decreased VEGF levels by the concurrent use of celecoxib with FOLFIRI [23]. When compared to sporadic forms, COX inhibition has shown statistically significant results in lowering the risk of familial CRC [4].

This case-control study has studied the effects of selective and non-selective COX-2 inhibitors on colon cancer risk [14]. Selective COX-2 inhibitors, regular aspirin, ibuprofen, or naproxen all significantly reduced risk, according to the findings. Acetaminophen and low-dose aspirin did not significantly alter colon cancer risk. These findings highlight the potential of selective and non-selective COX-2 inhibitors for the chemoprevention of colon cancer [21]. Venkatachala et al. conducted a cross-sectional study using 65 instances of CRC. The malignancies were classified as low or high positives according to overall COX-2 scores and were linked with clinicopathological characteristics. Overexpression of COX-2 was linked to a deeper invasion (p = 0.021), a higher stage of the tumor (p = 0.05), a higher rate of spread to lymph nodes, and less differentiation. This shows that the link between COX-2 overexpression and rising degree and depth of invasion may support using COX-2 inhibitors as a chemo and radiation adjuvant [14].

Cardiovascular Events Linked to COX-2 Inhibitors: A Therapeutic Roadblock

In a randomized, double-blind, placebo-controlled trial involving 1,561 subjects who had previously undergone adenoma removal, daily treatment with the COX-2 inhibitor celecoxib at a dose of 400 mg decreased detection through year three by 35.7% [10]. In the celecoxib group, the cumulative rate of advanced adenomas was also markedly decreased. However, the celecoxib group experienced major cardiovascular events slightly more frequently than the placebo group (2.5% vs. 1.9%, RR = 1.30, 95% CI = 0.65-2.62) [17]. In a study of 2,586 individuals with colorectal adenomas, rofecoxib use (25 mg daily) was associated with an increased risk of thrombotic cardiovascular events. Over 3,059 patient-years, 46 rofecoxib-treated patients experienced events compared to 26 patients in the placebo group. Mortality rates were similar in both groups [18]. Moreover, 2,587 individuals with a history of adenomas participated in a trial by Baron et al., and they were randomly assigned to receive either a placebo or 25 mg of rofecoxib [19]. When compared to placebo, rofecoxib significantly reduced the recurrence of adenomas (41% vs. 55%). In the first year, the protective impact was more noticeable. However, rofecoxib was linked to higher risks of GI and cardiovascular problems [19]. In conclusion, even though rofecoxib reduced the risk of adenoma, its possible toxicity needs to be taken into account. However, Ye et al.'s investigation indicated that the number of cardiovascular events had not significantly increased and that only one out of five studies had to be stopped due to a marginal increase in the number of cardiovascular events [26].

Ma et al. conducted a systematic review and meta-analysis of 32 randomized controlled trials involving 278,694 participants. The results showed that cyclo-oxygenase inhibitors significantly reduced the risk of colorectal, advanced adenoma, and metachronous adenoma compared to placebo, but they also increased the risk of serious side effects. Other interventions, including low-dose aspirin, folic acid, ursodeoxycholic acid (UDCA), vitamin D, and calcium, did not significantly reduce adenoma risk. They concluded that the evidence does not strongly support the regular use of COX inhibitors or low-dose aspirin for colorectal adenoma prevention [20]. Although using low doses of aspirin is linked to a lower incidence of cardiovascular events, the effectiveness of this medication in preventing CRC is constrained by extremely variable outcomes caused by the length of follow-up, the duration of usage, and the timing of outcome evaluation [11].

Limitations

It is worth noting that our review has certain limitations. Firstly, the available evidence is primarily based on observational studies and a limited number of randomized controlled trials. This may introduce bias and affect the strength of the conclusions drawn. Additionally, variations in study design, dosages, and duration of COX-2 inhibitor use make it challenging to establish consistent recommendations. Further well-designed randomized controlled trials are needed to validate the findings and determine the optimal dosage and duration of COX-2 inhibitor therapy for CRC prevention in genetically predisposed individuals.

## Conclusions

This systematic review explored the long-term usage of COX-2 inhibitors in treating CRC in most genetically predisposed patients, particularly in patients with Lynch syndrome and familial adenomatosis polyposis. Based on the articles reviewed, we found that COX-2 inhibitors are protective against colorectal malignancy. Early recognition of COX-2 m-RNA helps improve the prognosis of the disease. However, the evidence is limited and conflicting. Long-term usage of COX-2 inhibitors has been a topic of debate and research, especially regarding their effects on cardiovascular health. While these drugs have shown promise in reducing inflammation and preventing the growth of cancer cells, the risk of cardiovascular events associated with their use cannot be ignored. Therefore, using COX-2 inhibitors should be carefully considered on a case-by-case basis, considering the patient's medical history and risk factors. Healthcare professionals should closely monitor and encourage patients to report any new symptoms or changes in their health status while taking these medications. We believe further studies are needed to confirm the protective effects of COX-2 inhibitors on colorectal malignancy incidence in genetically predisposed individuals, to identify the optimal duration and dose of COX-2 inhibitor usage for chemoprevention, and to minimize the risk of adverse events.
